# Academy of Stomatology

**Published:** 1901-03

**Authors:** 

**Affiliations:** Academy of Stomatology


### Reports of Society Meetings.

### ACADEMY OF STOMATOLOGY.

A REGULAR meeting of the Academy of Stomatology of Philadelphia was held at the rooms of the Academy, 1731 Chestnut Street, on the evening of Tuesday, December 28, 1900, the President, Dr. J. T. Lippencott, in the chair.

A discussion was held upon the paper read at the previous meeting in November, by Dr. W. Leon Ellerbeck, entitled "Instrumental Precision in Dental Practice: A Review of some of the Investigations of Dr. G. V. Black."

(For Dr. Ellerbeck's paper, see page 1.)

### DISCUSSION.

*Dr. Curry.*—I have not very much to say, but this seems to be a case where the "last shall be first." The length of time that has elapsed since the reading of the paper obscures many points, though I listened attentively. I think that we must all agree that the preparation of the paper required a great deal of work, and I am glad of having had the pleasure of listening to it, but I believe many here will differ from the essayist in a number of his conclusions.

For myself I must say, it is very hard for me to believe that soft teeth are hard and hard teeth are soft,—that is, that all teeth are alike as regards their conditions. I do not think there has ever been any argument as to the percentage of organic and inorganic materials, but I think we all recognize that there are good teeth and poor teeth as we speak of them clinically, and we agree that the treatment ought to be according to our judgment based on clinical experience, not according to the conditions which reveal themselves to the chemist in his laboratory. I presume the brain of an idiot and the brain of a sage would be practically the same in a chemical analysis, and yet they would certainly deserve different treatment in the individual.

I do not think that it is necessary to condense gold fillings solidly all the way through. I do not believe it is even good treatment in all cases. Fill the cavity half-way with soft gold and finish with cohesive. I found in the mouth of a patient, a gentleman of sixty-five years, a soft gold filling which had been doing service for forty or forty-five years. It had been put there by a travelling clock-tinker. I could stick an excavator in it anywhere, but it was saving the tooth, and I left it there.

I have never seen an amalgam filling crushed in the tooth, and I have seen fillings that have been doing service for a great many years, which were made of very poor material.

In his original paper Dr. Black says he finds the phagodynamometer unsatisfactory in testing the strength of the jaws. If so, I do not see why so many accurate scientific conclusions can be based on its workings. I do not see how, from a clinical standpoint, Dr. Black's papers have been "a godsend to the dental profession."

I would like to ask the essayist if he has ever seen the poorest amalgam,—that is, what he would call the poorest,—made from the worst formula he knows, crushed in a cavity that had been properly prepared and filled.

*Dr. Register.*—This is an extremely interesting subject. I have read Dr. Black's work with a great deal of profit, and compared it with that of some of his predecessors, who have not given us the same experimental explanations. I think, however, that scientific men, in following up the pursuit of special study, discover a swallow and think they have found a summer. Dr. Black may see more than one, but the summer of perfect knowledge on this subject is not yet. There is one part of Dr. Black's experiments that impressed me more than all others, and that was that the analysis of tooth-structure is the same in all the different stages of life. I think that impressed me more than anything else that Dr. Black made known. I have given the subject a little attention since, and the information I can obtain seems to imply that the tissues of the body are all that way, whether the individual be young or old, weak or strong; the analysis of the part varies little. We know the physical conditions are not the same, they running through the whole gamut of potential energy. Now, should not this difference in physical manifestations, as clinically observed, claim our attention and guide our practice, rather than results secured from devitalized conditions, no matter how perfect they may be? A neurologist of this city, in speaking of the teeth from a diagnostic point of view, said that the subject had interested him, because he had noted in many brain and nervous troubles the nutrient or trophic nerves presaged the consummation of certain conditions; for instance, in such a condition as locomotor ataxia, several years before the individual would be fully affected the teeth were among the first to show evidences of the coming dissolution. May not this trophic influence be credited with gum and root diseases, particularly recessions? Dr. Black made a remark concerning alloys that impressed me very much. He said that in copper amalgam there is a fixed condition, not subject to change. Now, that is the one alloy which we know to be a true chemical compound, and when it is brought into juxtaposition with dentine prepared to receive it we know through clinical observation that this statement is a fact, and copper alloy is the one chemical compound we have to-day, all other alloys we are using being mechanical mixtures. May not the flow he lays so much stress on be due to the natural tendency of the amalgam and the character of the chemical union of the alloy and mercury? To remove oxidation that exists and bring the components of the mass into actual contact at the time of using, alcohol washing, although not new, is strongly urged, as making the molecular union more absolute.

Fillings thus washed not only present a much better appearance than those that are not so treated, but the operation bespeaks tooth preservation. I think there is a great opportunity for an alloy that will have the advantages of the copper amalgam without its disadvantages. I am using one that is a close running-mate, and Dr. Black is its author.

*Dr. C. N. Peirce.*—The paper to which we had the pleasure of listening a month since was certainly a very carefully prepared review of Dr. G. V. Black's investigations, and to the essayist of that evening we are most emphatically indebted for his painstaking effort. But to commend the author of the paper read on the 26th and to criticise Dr. Black's work and deductions are two widely different functions.

With Dr. Black's microscopical work every dental student has for years been somewhat familiar, and with pride and pleasure has the speaker many, many times referred to his unsurpassed technique. Certainly no one has done more accurate and valuable work than Dr. Black in the preparation of sections of teeth.

In the papers under discussion the doctor abandons the instrument, with which he was as familiar as a child is with his toy, and brings to the dental profession a series of papers on the analysis of tooth-structure and material for tooth-filling,—deductions based on an "instrument of precision," this instrument differing widely in its construction and manipulation from the microscope, and I think I may add, far more unreliable in its demonstrations.

I shall not attempt to respond in the order in which the essayist presented the salient points in the papers, but rather in the order of their importance. Says the essayist of the evening, "It was conclusively proved that there was no withdrawal of lime salts from the teeth of women during pregnancy."

Does not the author of the paper recognize this fact, that the law of reproduction is that the embryo shall be nourished even at the sacrifice of the mother, and that while thousands of mothers bear children without any nutritional disturbance, there are other thousands who suffer gastric disturbance and other functional derangements, which are in their effects a serious tax upon the nutrition of the mother? So with this condition prolonged for months the embryo is feeding, fattening, and growing at the expense of the mother. Specialists in obstetrics recognize this when they state that the bones and teeth, as well as the soft tissues, suffer—I will not say in their density, but in their bulk and conformation—during this period of gestation. The essayist, or, rather, the author for whom he is speaking, makes one vital error when he assumes that the decalcification of the tooth necessarily lessens the density of what remains. Not so at all. The lime is carried from that part which is in close contact with the vascular pulp, and if Dr. Black has had the same experience that some of us in Philadelphia have had, he would never have made the assertion that he conclusively proved that there was no loss of lime from systemic conditions. Any observing dentist who has had nearly fifty years of active practice has seen pulp-chambers enlarged, as well as lessened, in quite a limited period. Therefore the statement that there is no withdrawal of lime salts from teeth during pregnancy or any other condition of life, is made from limited knowledge and hence is not authority. Of variations in percentage of lime salts this remarkable statement is made: "The greatest variations were in the different teeth in the same individual." Remarkable, because it could not be otherwise. Does not Dr. Black, as well as his representative, appreciate the fact that the large majority of individuals use only one side of the mouth, just as they use one eye or one ear? Use and disuse come in as an important factor, and though the difference was ever so slight in density of structure, it would, without any doubt, be exaggerated in that locality where the function of the pulp was the active agent in the change. "The elasticity of dental tissue did not depend upon the density of the teeth, or upon the percentage of lime salts, yet it apparently varied somewhat according to the condition of the organic matrix." This is a very important admission, and it is the key to the predisposition of the teeth of the young to decay. His instrument of precision may or may not demonstrate the density of the teeth of children. What I do know is that the organic matrix, for want of use, is not performing its normal function, hence from the absence of this continuity which should exist between the mineral and organic structures the teeth yield to the ravages of decay, which can (I speak advisedly) be checked, if not entirely arrested, by the function of mastication being fully performed.

"The presence or absence of caries is in no way a factor in determining either the hardness or elasticity of tooth-structure or *vice versa.*"

Probably this is true, but Dr. Black has prepared sections of dentinal tubuli to but little personal instruction if he does not know that the slow progress of dental decay will, without any doubt, increase the amount of lime salts not only in the tubuli, but also on the periphery of the pulp to the protection of that organ from exposure at the point to which the decay is progressing.

"The prevailing idea of white teeth being soft was found to be an error, an example of such resisting a strain until the application of three hundred pounds."

What has the resistance of three hundred pounds to do with the experience of thousands of dentists? The arches formed by the tubules may resist twice that strain or force, but this statement will not interfere with the fact that the progress of dental decay meets with less resistance in white teeth than in the majority of yellow teeth. The facts are adverse to Dr. Black's deductions, and from our present knowledge the teeth themselves are responsible for the facts.

"The active cause of caries is a thing apart from the teeth themselves, acting on them from without." This is probably true, for the enamel possesses but a trace of organic matter and but slight circulation, but if from this the inference is to be drawn that systemic conditions influencing subjacent tooth-structure is not a predisposing factor in the progress of dental decay, the evidence is against it. It is only necessary to recite one instance that has been the experience of many in practice. Attention by a patient is called to a certain locality in the mouth where condiments or sugar give a sensation of pain. On an examination the zonal line between enamel and cement may give evidence of a decided exalted sensibility. The transmission of touch to the brain is unmistakable, and unless great care is exercised decay results in a limited time. Now, the contention is that the predisposition is not wholly from without. Indeed, it is a question whether the previous or present systemic condition is not responsible for the exalted sensibility.

As the doctor's "instrument of precision" has fully proved that there is no perceptible difference in the density of teeth, he can well afford to say "that there is no basis for the supposition that calcic inflammation of the peridental membrane or phagedenic pericementitis attacks persons who have dense teeth in preference to those whose teeth are less dense." This point will not be contested, but the following is true: The large majority of teeth affected with "phagedenic pericementitis" are of yellow color, and at the time of the attack are, to a *limited extent only,* the seat of dental decay.

The experiments performed in the condensation of gold-foil, and the advantage of adhesive foil over soft, in the preservation of tooth-structure, are so entirely without practical value in tooth salvation that they are worth but little time in discussion.

That there have been more worthless fillings inserted with adhesive foil than with soft foil cannot be truthfully contradicted. That with hand-pressure in the use of both materials there can be as much gold in weight packed into a given space with soft as with adhesive gold cannot be denied, and that soft foil more readily adapts itself to the walls of the cavity is also true.

*Dr. Luckie.*—I am very glad that Dr. Black's articles that appeared five years ago are being reviewed. The review has been quite a long while in coming. The society should be congratulated in having a member who can make these tests, as demonstrated at the last meeting, and I think it would be well if the fillings introduced into the steel matrix were made by different members of the society and their strength tested here. Dr. Peirce makes the assertion that he can put more soft gold than cohesive gold into a given cavity. It might do well to test the strength of that gold. He speaks of being a lover of soft gold. I am a lover of soft gold, and use more soft gold than cohesive gold. I would like to have some of my fillings tested. With soft gold you can make contour fillings, and they are apparently as strong in the mouth as cohesive gold. I think it would be a good thing to continue the experiments in that direction and have further discussion.

*Dr. James Truman.*—Mr. Chairman, I did not care to talk on this subject to-night, for the reason that I have written a good deal upon it, and, therefore, it places me in the position of repeating myself. At one time, five years ago, when Dr. Black presented his series of papers, I, with a number of others, replied to them and criticised them, and among replies that I made at that time were that Dr. Black in making his experiments failed, so far as my understanding went, to so explain the beginning of his experiments that we were left in doubt. He stated that all teeth had the same amount of density, that there was no such thing as soft teeth as was understood by the profession generally, and hard teeth, but all teeth had the same amount of calcific matter. I then stated that, in my opinion, the method that he adopted to secure teeth was open to criticism; that he had individuals send him quantities of teeth taken from various mouths and of all ages, but without any regard to the character of the teeth, as this has been generally understood. He placed all those teeth under experimentation and consequently the result was as he stated. Now, as I understand it and as every one else who has practised dentistry for many years understands it, there are teeth that are soft and chalky, that cut under the instrument almost like cheese, and others that present great resistance to cutting force. The environments do not seem to have much to do with this. Now, those soft, chalky teeth are not to be had extra-orally. I do not suppose any experimenter could possibly secure a sufficient number of that character of teeth with which to make laboratory experiments. Dr. Black says they are white teeth. I do not observe that they are white teeth; they are more like mother-of-pearl. What was his answer to that? He says that Dr. Truman was the only one of all his critics to make that criticism. That may have been true; so far as I know, it was true; but does that invalidate the criticism at all? Not as I view it. He failed to reply to this objection. Therefore I hold that his experiments at the very start were defective and lead to wrong conclusions.

Another point relates to his instruments. Does any one know that they register with absolute accuracy? Dr. Black does not prove it. He says his different instruments give certain readings, and we are supposed to accept those statements as absolutely correct. Now, I, for one, am not prepared to accept them at their face valuation. There are some statements made in his paper that to my mind are impossible of belief. In a recent paper, published in the December number of the *Dental Cosmos,* and read in October before one of the New York State societies, he goes over a good deal of the same ground, and says, among other things, that the saliva is acid. He does not say it in so many words, but by implication, because he quotes from the statements of others and seems to endorse them.

What do we mean by the saliva?

It seems to me there is a great mistake made by men who talk about the "oral fluid" being the "saliva." It is not saliva; that is, it is not wholly saliva. The saliva, from my very earliest recollection and knowledge of dentistry, has always been stated as being alkaline. I make a distinction between the oral fluid and the saliva proper, as the latter enters the mouth from the ducts. I have been testing mouths for many years, and I have never yet found an absolutely acid condition. The oral fluid is neutral, and it was this reaction that led to experiments at night to find whether any change took place then in a condition of rest, and I invariably found an acid reaction during the night. This is now generally accepted, that the night season is the time for acid production. I never, however, received any credit for that work.

In regard to Dr. Black's force in mastication, is there any one who can accept his gnathodynamometer readings? He mentions that the force of two teeth coming together is probably equal to three hundred pounds. Does anybody believe that?

I did not feel like talking to-night, because I do not regard it as proper to answer a man who has secured certain results through laboratory work without going through a similar series of experiments. After hearing Dr. Ellerbeck's very excellent *résumé* of Dr. Black's paper, I had some idea of having an instrument manufactured for the purpose of repeating these experiments. But I found the time was too short before the present meeting to accomplish anything, so that I am obliged, like the rest of you, to come here to-night and talk from clinical observation, or, rather, from common-sense observation.

A recent writer in the INTERNATIONAL DENTAL JOURNAL, Dr. B. F. Arrington, of Goldsboro, N. C., was so impressed with these statements of Dr. Black's that he engaged a boy to crush hazel-nuts and lemon-drops, as described by Dr. Black in his original article. He kept him six minutes crushing one hundred and fifty lemon-drops, and the same time crushing hazel-nuts, and, if Dr. Black is correct, it took a force for each hazel-nut of one hundred and forty-nine pounds, and sixty-five pounds for each lemon-drop. The total of that boy's twelve minutes would be over thirty-two thousand pounds. Just think of it, thirty-two thousand pounds of force in twelve minutes! Is there not something wrong about Dr. Black's gnathodynamometer? Just think of such a force used by a child! And he makes no exception. He says, from one hundred and fifty to four hundred pounds crushing stress between two teeth. Dr. Arrington tried to have the boy lift a weight of seventy pounds. He could not do it, and yet he was crushing, according to Dr. Black's article, at the rate of probably three hundred pounds every time he brought his jaws together.

Now, is it reasonable that the pterygoid and masseter muscles should be able to do more than that child, or than an individual could, in raising the weight, when all the muscles of the body are in force at the same time? You know what a strain it would be to lift three hundred pounds.

The whole thing to me, without testing, seems absolutely impossible of belief. I cannot accept it until I first know that the instrument that is used to demonstrate this has been thoroughly tested as to weight. I do not know whether Dr. Black has done this or not. If he has, there is probably some error connected with it.

A matter has been brought up here which is hardly germane to the question, that of filling with soft foil. I, like Dr. Pierce, was trained in soft foil. There was nothing else in our earlier days, and we thought we could make gold fillings fairly solid. I question, however, whether it is possible to pack in a matrix soft gold-foil of the old style as solidly as you can pack cohesive gold with an equal degree of care. I do not want to enlarge on that; it does not particularly belong to the question at issue. I tested, some thirty years ago, this whole matter of filling, and had all the fillings weighed at the United States Mint, and I think I know something about the difference between hand-pressure, pressure upon soft base, and condensation made by the electric mallet.

*Dr. Roberts.*—There have been many points brought up in this discussion. Referring particularly to the question, Is the enamel a lifeless or a live tissue? If it is a live tissue, and receives nourishment after it is once formed, then systemic conditions may affect it. If not, they can have no effect upon it. The question, then, is purely one of environment. Whether the tooth is hard or soft can make no difference. It is simply that the environment is changed, according to the systemic condition. I think the statement was made that the pressure for packing amalgam was fifteen pounds. Is this meant to be fifteen pounds to the square inch, or fifteen pounds to the one-hundredth of a square inch? With regard to the density of your filling, it depends very much upon whether the cavity has four walls and a bottom or whether it has not. The soft or cohesive foil, that can be almost perforated with an instrument, if placed in a cavity such as you get in a steel matrix, cannot be crushed, though not very solid. There is only one way possible to make it give or break, and that is by forcing something smaller than the cavity into your matrix or tooth. I have seen in the mouth amalgams which you cannot call crushed, because they are not crushed. They are rubbed down, worn down, mashed to one side, rolled out, hammered out, if you choose; they are not crushed to powder. I believe the weakest amalgam that you can get that does not disintegrate in the mouth, as the copper amalgams will, is amply strong for all cavities in the mouth, excepting where contour fillings of great strength are required. The work of Dr. Black, I think, has been of great benefit to the profession. In some directions it has brought out thought. It has brought out the proper formation of cavities better than anything else has ever done. The enamel is practically valueless to hold or retain the filling. I am very glad that Dr. Ellerbeck was able to give us so complete a *résumé* of Dr. Black's work, and I thank him for his paper.

*Dr. Ellerbeck.*—I have prepared myself only as the discussion has advanced, and would not speak but that statements have been made which, if permitted to go unnoticed, might create injustice to Dr. Black. It seems that in the main those most vehement in the denunciation of his work are they who have given it the least study. Those next in error are they who look upon it with biassed minds, and give but little credit for practical value to Dr. Black's work as a whole, preferring to note a few points of minor importance at variance with their own beliefs, and, not accepting these, denounce the results of his patient efforts as of little or no importance to the dental world. Dr. Black is made to say things which, as a matter of fact, he does not say or believe. It is common to hear that he believes the teeth of the youth and the teeth of the adult to be of the same density. In fact, Dr. Black's belief distinctly is that teeth of different ages *do* differ in density, so slightly, however, as not to account for the tooth-tissues in themselves being responsible for any predisposition to assume pathological conditions.

The main line of argument to-night has been the expression of individual opinions based upon clinical evidence alone. While it would be hardly proper to say that the observations which the gentlemen have cited are wholly wrong, still, as opposed to them we must not overlook the fact that Dr. Black has had some forty or more years of active practice, and no one perhaps in the profession has been more keenly alive to clinical evidence than he. Perhaps no one has kept more careful records. Along with this he has instrumental evidence in support of his conclusions, and while not overlooking the fact that we may at times be right, the mere unsupported statement that we do not believe is not sufficiently scientific to have any weight as argument. It is claimed throughout a certain section of the country, where the later principles of operative procedure are more generally in vogue, that the clinical evidences are clearly vindicating the instrumental evidence and better operating is being done. The clearest proof of the truth of principles is in their successful practical application.

As to the resistance of teeth to cutting instruments, let any one prepare slabs of dentine free from carious infection, and from different teeth clinically known as hard and soft, and, having mounted them in a way that they may be firmly held, let him close his eyes and, not knowing which slab he has selected, endeavor to distinguish between the different specimens by their resistance to cutting instruments, using for the purpose sharp, square-end hoe excavators. The number of errors he will make in a hundred trials may convince him that the difference is not so great as to be readily appreciated by tactile sense.

Dr. Black's argument is almost wholly along the line of a systemic influence having to do with the beginning of the carious process, something operating through the oral fluids or saliva being the essential predisposing cause. I specify saliva in answer to the remark of one of the gentlemen questioning Dr. Black's use of the term oral fluids, for, whether it is confined to the saliva or not, the point is hardly well taken, for certainly the saliva would contaminate the oral fluids, and surely this would not render the predisposing causes inoperative.

As to the statement that Dr. Black does not believe in the administration of medicinal agents for the prevention of decay, I have observed no statement of his to this effect. On the contrary, my understanding is that he argues the possibility of the administration of some antitoxin along the lines of serum therapy being the possible solution to the great question of prevention of tooth decay. From the recent experiments of Michaels, of Paris, it seems that Dr. Black's suspicions are to be realized, and that the predisposing cause of caries is to be found in the saliva, which is contaminated in varying quantities with histo-chemical products, due to physiological changes accompanying various diethetic conditions, the chemical products in turn influencing bacterial growth. Where the gentleman has perhaps gotten his misunderstanding is in Dr. Black's statement that he does not believe that the administration of lime in solution with a view of preventing the withdrawal of calcified matter from the teeth of pregnant women, or of producing better calcified teeth in the offspring, is of any value. Dr. Black, of course, arrives at this conclusion because of his determining that there is no withdrawal of lime from the teeth of pregnant women in any event, and while he offers no objection to the administration of lime-water as a neutralizer to hyperacid conditions, he thinks the claims made as to its possible physiological assimilation are absurd. While organic substances containing calcium salts in complex molecules may tend to furnish the lime for both bones and teeth, its assimilation only affects the rapidity of the calcification and not the extent.

One of the gentlemen stated that the brain of an idiot and the brain of a sage, whilst of the same chemical composition, would certainly deserve different treatment.

Now, if we were to liken the different brains of the individuals to the pulps of teeth, and fractures of the skull as like unto carious cavities, I believe the treatment by surgical trephine would in both cases be conducted similarly.

I call attention to the argument that since amalgam fillings rarely crush under the force of mastication, a filling which would withstand two hundred and forty pounds pressure can with impunity be used, and that there is no necessity for a higher crushing stress. It is about as sound argument to say that because we can subsist on a milk diet there is no occasion for better food, since this will maintain life. We are in duty bound to render patients our best service, .nd while a filling which would withstand a pressure of two hundred and forty pounds without crushing might be a continual menace because of its liability to flow, with high crushing stress we have a minimum degree of flow, from which it is evident that high crushing stress is not the only requirement, but, while being of considerable advantage, it is the best means of guarding against the disadvantages of flow. While perhaps not always true, as, for instance, where the modifying metal induces weakness, low crushing alloys contain too high a proportion of tin, consequently, besides their weakness, they usually shrink perceptibly. Some of the high-class alloys crush at approximately four hundred pounds, but flow only from one-half to one per cent., expanding a point or two at most; whilst some alloys, among them certain gold and platinum alloys, which have been sold in large quantities throughout the country, crush at approximately two hundred and forty pounds, flow from ten to twenty per cent., and shrink ten points. As these high-class alloys and low-class alloys sell at approximately the same price, and the attributes are all in favor of the high-class alloy, except ease of insertion, it is evident that we should employ them. True, they require considerably more manipulative ability for their insertion and considerable practice to obtain the best results, but we cannot excuse ourselves because of lack of manipulative ability. I believe that Dr. Black's latest experiments go to show that twenty pounds pressure with a special plugger is required to produce the best results. The form of the plugger he is modifying in accordance with his findings.

In relation to copper amalgam, I think the gentleman who made the statement that copper is the only metal to unite with mercury chemically would find some difficulty in supporting his opinion. There is some conclusive proof that gold and silver unite with mercury in atomic proportions; in fact, the definite chemical compounds of each may be isolated.

One of the gentlemen argues that he can make as dense or a denser filling with soft gold than can be made with cohesive. Therefore he does not hesitate to make the bulk, if not all, of the filling with this material. In non-cohesive gold the individuality of the leaves obtains, and there is no cohesive condensation through intermolecular contact, as would appear to be the case with fillings made of cohesive foils. Besides, even were it possible to obtain the same density with soft foil as can be obtained with cohesive, this, while necessary, is only of even importance as regards fillings in occluso-proximal cavities, for here there is much wear, and rigidity, which cannot be obtained with soft foil, is of great importance. While there is no objection to a thin lining of soft gold, it should certainly be used only in small amount, lest it materially weaken a filling much exposed to wear. One of the experiments conducted here before the Academy at the last meeting shows to some extent the difference between non-cohesive and cohesive gold as regards resistance to crushing stress. A filling 0.08 of an inch cube, largely non-cohesive gold, finished with cohesive,—such a method as suggested by the gentleman as being his preference,—shortened approximately twelve per cent. under a pressure of two hundred pounds, while one of the same dimensions, made of cohesive gold throughout, shortened only four per cent., though it has been determined that a *thin* lining of non-cohesive gold seems to make but little difference. So, if we wish to use any soft gold with a view of securing better adaptation at the cervix, it is well to limit the amount.

One of the gentlemen states that he can see no practical value in Dr. Black's work. I trust that a further study of it may be more convincing.

Dr. Black's system of cavity preparation in accordance with the principles of physics, and his methodical procedure in their preparation, savoring of accuracy and despatch; his determination of the strength requirements of the filling-material to the strength of teeth and strength of bite, and the best means of securing this strength, both in amalgam and gold; and, withal, his system of nomenclature, which enables us to communicate understandingly methods of procedure and exact tooth areas operated on without necessity for diagrammatic explanation, must appeal strongly to the scientific; and while there may be some weak points, its universal adoption until something better is presented would be of benefit to the whole profession, since it would reduce methods of procedure to a systematic and definite basis.

There are many things about which I would like to speak, but I feel that I have about used up my apportionment of time. One thing of interest, and of which I will speak, however, is of the experiments with gold fillings inserted in a steel matrix. The cube cavity is formed by three pieces of steel, accurately ground together and clamped. In filling this cavity with gold, using the amount of care usually bestowed by careful operators, it is found that, notwithstanding the accurate fit of the different pieces of steel used to make the cube cavity, the gold will flow out into the crevices, filling the angles perfectly. How often we have heard the statement, Do not attempt to fill into an angle. As a matter of fact, it is found that we can fill perfectly a cube in which there are many angles, and this is of interest because a square is the most retentive form of cavity, and while we would round out angles in enamel margins, the method of preparing in molars and bicuspids a flat cervix to keep the gold from condensing upon itself, together with a square arrangement of the cavity, in itself retentive, and in addition an anchorage in the occlusal portion to prevent tipping, is scientific, presents the attributes of ease of insertion, and undoubtedly adds durability to the filling. It is necessary, in order to make a groove, cut with a round bur, retentive, that it be sunk in the tooth-structure over half its diameter; whereas, in the method of using small, square-end hoe excavators to make the under cuts, as, for instance, in weak incisors, the grooves made, however shallow, are retentive. Their proper placement along the palatal and labial dentinal walls, as compared with burred grooves, is conservative of tooth-structure, and they are equally if not more retentive, and we have learned that they can be perfectly filled.

One of the gentlemen has questioned the accuracy of Dr. Black's instruments, particularly the gnathodynamometer used in determining strength of bite. He says he is of the belief that the jaws are incapable of exerting a force of three hundred pounds, and cites in proof an experiment conducted by Dr. Arrington, of North Carolina, who hired a boy to crack hazel-nuts and lemon-drops breakable respectively at one hundred and fifty and sixty-five pounds, and found that the boy in the short space of twelve minutes cracked one hundred and fifty hazel-nuts and one hundred and fifty lemon-drops, which, multiplied by the stress required for each, would, as "claimed and proclaimed by the converts and advocates of the phagodynamometer," be equivalent to about thirty-two thousand pounds,—sixteen tons of work done in twelve minutes,—which he thinks physiologically impossible and absurd. As a matter of fact, at least some "advocates and converts of the phagodynamometer" do not get so far astray on their understanding of the laws of mechanics as has Dr. Arrington, and are able to distinguish between work done—foot-pounds per minute—and static pressure, which latter is the simple but conclusive explanation of how the converts and advocates of the phagodynamometer do explain the physiological phenomena.

To them the physiological possibility is as easily apparent as Dr. Arrington, whose experiments have been cited, thinks it difficult to conceive. In pressing down a pound or a fraction thereof more than is necessary to crush a nut or a lemon-drop, much energy as to work is not necessarily expressed. Internal work in maintaining static pressure, as, for instance, in cracking a nut, is not expressed in foot-pounds, but is almost wholly expressed in heat. However, why compare the strength of the different body muscles when some are designed for one purpose and some for another, some for work and some for maintaining static pressure? And, too, there is evidently a quality in muscle according to its arrangement,—length and breadth of fibre and the effectiveness of the levers which these muscles operate. On the one hand, a flea can jump many hundreds of times his length; his bite is also effective, but he would find locomotion difficult were he hitched to a pin. Again, a crab can exert a force with his claws so great that our whole efforts are necessary to secure our freedom when once within his grasp (static pressure and analogous to strength of bite); yet the crab weighs but a pound or two, and while not a scientific comparison, but analogous to Dr. Arrington's reasoning, a crab could not move a brick to save his shell. While not confusing foot-pounds with static pressure, and not overlooking the different purposes for which men and crabs were intended, comparing the size of a crab, his ability to perform work, and claw strength with man and his ability for work, what might we not expect man's strength of bite to be? Three hundred pounds is but the degenerative result of a higher civilization, and no doubt low compared with the jaw strength of the preadamites.

As for the phagodynamometer being too inaccurate to warrant scientific conclusions being based on its workings, all evidence goes to show that if it is inaccurate it does not register the full force of which the jaws are capable, for the peculiarity of its construction necessarily keeps the jaws apart, and it operates therefore at a disadvantage.

In conclusion let us consider the exception taken to Dr. Black's argument that there is no basis for the statement that phagedenic pericementitis most frequently attacks dense teeth. The gentleman who takes issue with Dr. Black's view states that he has examined perhaps as many cases of phagedenic pericementitis—so-called pyorrhœa—as any man living, and that he has found in the great majority of cases that dense yellow teeth of a bilious type have been the ones affected. The gentleman offers no proof that the teeth are denser as regards percentage of calcium salts or of a higher specific gravity. On the other hand, Dr. Black arrives at his conclusions thus: The average age of individuals whose teeth are being lost through pyorrhœa is fifty years. The average density as regards lime salts of all teeth at the age of fifty years is 63.83. Now, the average density of teeth lost through pyorrhœa is within an inappreciable difference of a fraction of a per cent. the same. In other words, the density is only that wrought through the natural process of time, and normal for the age of the patient, having neither an undue percentage of calcium salts nor unusual specific gravity. So we cannot state that pyorrhœa attacks these teeth because of their excessive density. While not denying the bilious type as constituting the majority of teeth affected, let us look to another source for the cause of the affection. It is being found that diathetic conditions are largely responsible for the predisposition of teeth to decay. Diathetic conditions especially characteristic of the type or temperament mentioned by the gentleman may be largely responsible for a predisposition of the tooth membrane to become the seat of infection, thus inducing the pericemental disease.

Now, it seems that, so far as any argument or proof presented to-night is concerned, Dr. Black's experiments and conclusions remain unshattered. Let us not attempt to disparage scientifically deduced opinions unless we can present better ones similarly deduced, for by so doing we may unwittingly assist in the suppression of truths which might be of great benefit to ourselves, the rising dental generation, and the body politic.

Otto E. Inglis,
*Editor Academy of Stomatology.*

